# FACT: Feature Aggregation and Convolution with Transformers for predicting drug classification code

**DOI:** 10.1093/bioinformatics/btaf184

**Published:** 2025-07-15

**Authors:** Gwang-Hyeon Yun, Jong-Hoon Park, Young-Rae Cho

**Affiliations:** Division of Software, Yonsei University - Mirae Campus, Wonju-si, Gangwon-do 26493, Republic of Korea; Division of Software, Yonsei University - Mirae Campus, Wonju-si, Gangwon-do 26493, Republic of Korea; Division of Software, Yonsei University - Mirae Campus, Wonju-si, Gangwon-do 26493, Republic of Korea; Division of Digital Healthcare, Yonsei University - Mirae Campus, Wonju-si, Gangwon-do 26493, Republic of Korea

## Abstract

**Motivation:**

Drug repositioning, identifying new therapeutic applications for existing drugs, can significantly reduce the time and cost involved in drug development. Recent studies have explored the use of Anatomical Therapeutic Chemical (ATC) codes in drug repositioning, offering a systematic framework to predict ATC codes for a drug. The ATC classification system organizes drugs according to their chemical properties, pharmacological actions, and therapeutic effects. However, its complex hierarchical structure and the limited scalability at higher levels present significant challenges for achieving accurate ATC code prediction.

**Results:**

We propose a novel approach to predict ATC codes of drugs, named Feature Aggregation and Convolution with Transformer models (FACT). This method computes three types of drug similarities, incorporating ATC code similarity with hierarchical weights and masked drug–ATC code associations. These features are then aggregated for each target drug–ATC code pair and processed through a convolution-transformer encoder to generate three embeddings. The embeddings are finally used to estimate the probability of an association between the target pair. The experimental results demonstrate that the proposed method achieves an area under the receiver operating characteristic curve (AUROC) of 0.9805 and an area under the precision-recall curve of 0.9770 at level 4 of the ATC codes, outperforming the previous methods by 15.05% and 18.42%, respectively. This study highlights the effectiveness of integrating diverse drug features and the potential of transformer-based models in ATC code prediction.

**Availability and implementation:**

Source code of FACT is freely available at https://github.com/knhc1234/FACT.

## 1 Introduction

Drug development is considered a high-risk sector that requires substantial time and cost expenditure. Low success rates and escalating R&D expenses contribute to this high level of risk (Yang *et al.* 2019, Kim *et al.* 2022). The traditional drug development pipeline, which includes lead discovery, preclinical trials, clinical trials, and Food and Drug Administration approval, typically takes 10–15 years, with a low success rate of <10% (Krantz 1998, Parvathaneni *et al.* 2019, Luo *et al.* 2021). Given these significant challenges, drug repositioning, also known as drug repurposing, has become a progressively attractive alternative. Drug repositioning is the process of discovering new therapeutic applications for existing pharmaceuticals, including those already on the market or previously unsuccessful in clinical trials (Ashburn and Thor 2004, Oprea and Mestres 2012). This approach offers the advantage of accelerating the drug development process by bypassing several of the early stages, making it a faster and more cost-effective strategy (Park and Cho 2025).

In recent years, the use of Anatomical Therapeutic Chemical (ATC) codes has been increasingly recognized as a promising strategy to improve the effectiveness of drug repositioning (Das and Mazumder 2024). Developed by the World Health Organization, ATC codes provide an international system for classifying medications based on their chemical, pharmacological, and therapeutic properties. This system uses a five-level hierarchy to categorize drugs, supporting research and practical implementations in pharmacology and medicine. In particular, while a drug may have multiple ATC codes reflecting its various therapeutic effects, these codes do not account for unknown effects. This limitation presents an opportunity to leverage ATC codes to uncover novel therapeutic applications.

Previous research on predicting ATC codes for drugs has focused mainly on machine learning algorithms, especially deep learning, capable of handling large-scale data. These studies can be broadly divided into three main approaches (Chen *et al.* 2024): multi-label classification, binary classification, and other methods. The multi-label classification approach assigns a drug to multiple ATC codes within the same level. However, as the level of the codes increases, the number of categories grows substantially, introducing additional complexity and increasing the computational load on the model (Tang and Chen 2022, Yan *et al.* 2022). This limitation has resulted in most models focusing only on predictions at level 1 or exhibiting lower accuracy at higher levels. In contrast, binary classification addresses the presence or absence of specific ATC codes through binary decisions, allowing predictions to be made at all levels (Wang *et al.* 2013, Liu *et al.* 2015, Zhao *et al.* 2023). Lastly, there are various other methods, including network-based predictions and recommendation systems (Peng *et al.* 2021, Chen *et al.* 2024).

This study adopts binary classification due to its flexibility in predicting ATC codes at all levels. Dunkel *et al.* (2008) first proposed an ATC code prediction method by calculating structural similarities between compounds using molecular fingerprints. Wang *et al.* (2013) suggested generating kernel functions that take advantage of multiple measures of drug similarity and a single measure of ATC code similarity, which were subsequently used to classify drug classification codes with the support vector machine (SVM). Liu *et al.* (2015) integrated various drug characteristics, such as compound structure, target proteins, side effects (SEs), and drug-induced gene expression profiles, employing a logistic regression model to predict ATC codes. Zhao *et al.* (2023) advanced ATC code prediction by generating feature matrices from the structural similarity, the ATC code similarity, and drug–ATC code associations using a deep residual learning. Despite recent advances in deep learning methods, previous models still suffer from high variance and a significant decline in predictive performance at higher levels of the ATC hierarchy.

In this study, we propose a novel method, FACT, for predicting the ATC code of a drug. This method significantly improves both prediction performance and model stability compared to previous approaches. FACT generates three types of drug similarity matrices derived from chemical structures (CSs), drug–drug interactions (DDIs), and SE profiles. The model leverages feature aggregation from drug similarities, ATC code similarity, and drug–ATC code associations. Notably, instead of using the simple measures of ATC code similarity employed in previous studies (Wang *et al.* 2013, Zhao *et al.* 2023), we introduce the weighted-hierarchical similarity (WHS) of ATC codes, which assigns weights according to each level of the ATC classification, thereby further improving prediction performance. By utilizing convolution and transformers (Vaswani *et al.* 2017), FACT can effectively learn the associations between drugs and ATC codes, converting the aggregated features into final embeddings. These embeddings are processed through a fully connected layer followed by a sigmoid layer to estimate the association probability.

## 2 Materials and methods

**Figure 1.**
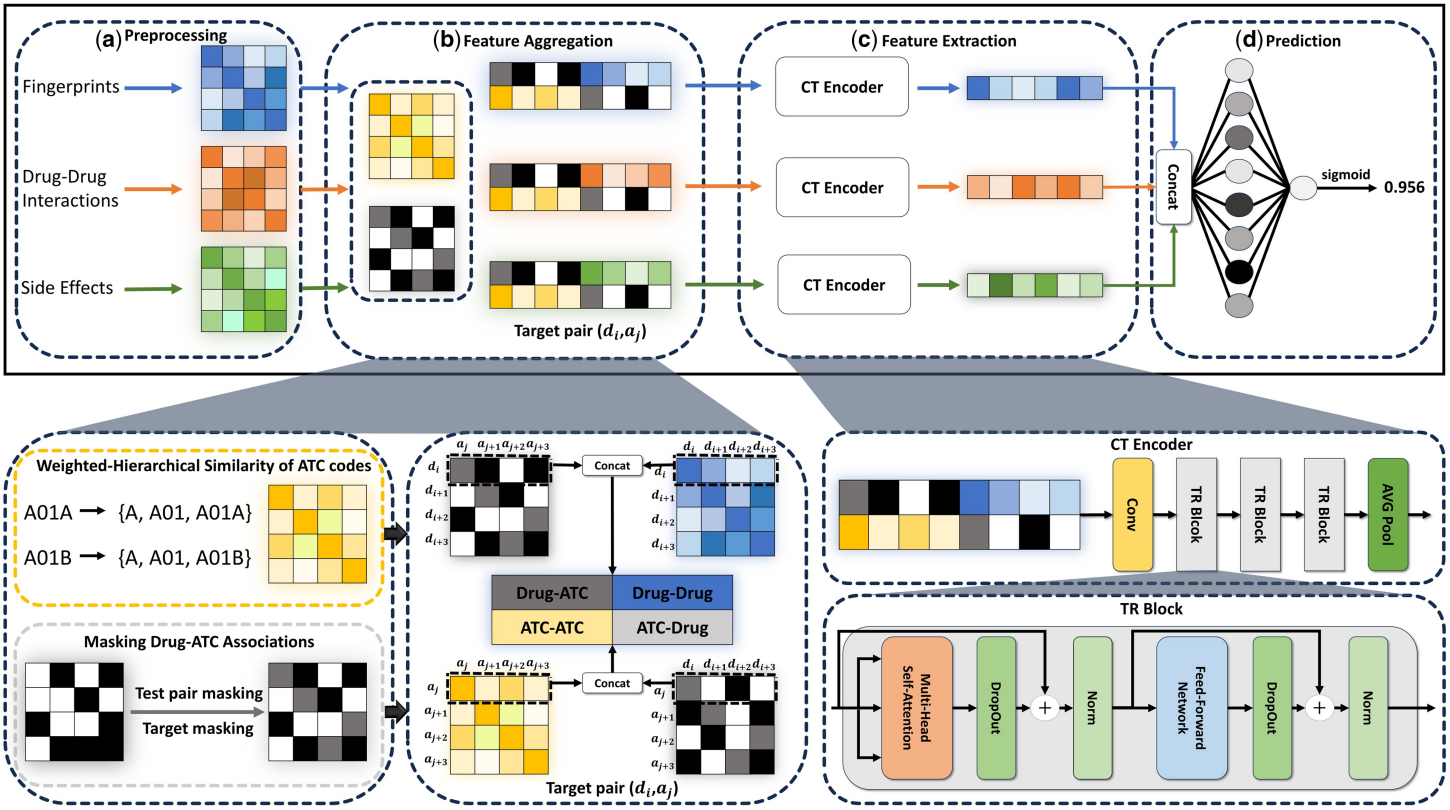
The overall structure of the proposed ATC code prediction model, FACT, is as follows: (a) First, it generates three drug similarity matrices based on drug fingerprints, drug–drug interactions, and side effects. (b) Next, an ATC similarity matrix is constructed by incorporating hierarchical weights and applying masking to drug–ATC code associations for the test and target pairs. The feature matrix for the target pair is generated by aggregating features containing relevant information. (c) Each matrix is then transformed into its final embedding through the CT encoder, which includes convolution, TR blocks, and average pooling. (d) The three embeddings are then processed through a fully connected layer, followed by a sigmoid layer to predict the drug’s ATC code.

The proposed framework, FACT, was designed to address the challenge of predicting ATC codes of drugs. As shown in Fig. 1, FACT consists of four stages: preprocessing, feature aggregation, feature extraction, and prediction. First, the preprocessing phase involves computing similarities derived from three characteristics of the drugs. These characteristics were selected because they are among the most widely used and empirically validated features in prior research. Second, in the feature aggregation phase, the drug similarity matrix, ATC code similarity matrix, and masked drug–ATC associations are aggregated for the target pair. Third, in the feature extraction phase, the aggregated features are processed through a convolution-transformer (CT) encoder to generate the final embeddings. Finally, in the prediction phase, these embeddings are used to predict new associations between drugs and ATC codes. Further details of each stage are provided in the following sections.

## 3 Preprocessing

### 3.1 Chemical structure similarity

The CS of drugs has been widely adopted to extract drug features in related studies. These approaches generally use the Simplified Molecular Input Line Entry System (SMILES) which contains information about atoms, bonds, rings, aromaticity, and branches in a computer-understandable format, representing the CS as a string of text. This SMILES data was converted into a chemical fingerprint, known as the Extended Connectivity Fingerprint (ECFP), a vector representation designed to analyze molecular properties and similarities. Subsequently, 1024-dimensional ECFPs were generated for the drugs. Using these fingerprints, CS similarities were calculated by the Tanimoto coefficient.

### 3.2 Drug–drug interaction similarity

DDIs refer to reactions that occur between two or more drugs or between drugs and food, beverages, or supplements. These interactions may arise when medications are taken in the context of specific diseases, which can lead to abnormal increases or decreases in drug efficacy and the occurrence of SEs. For instance, terfenadine, an antihistamine commonly used to relieve allergy symptoms, generally does not induce drowsiness when administered at appropriate doses due to its lack of central nervous system effects. However, when co-administered with ketoconazole, an antifungal medication, this interaction can significantly elevate terfenadine concentration, thereby increasing the risk of severe cardiovascular complications.

In addition to identifying potential risks, DDIs can also serve as a valuable resource for inferring drug similarities. If two different drugs exhibit similar effects with the same drug, it suggests that they may have similar functions. Based on this assumption, the similarity between two drugs can be calculated. In this study, we computed drug similarity using the proportion of shared interactions with other drugs, measured by the Jaccard coefficient.

### 3.3 Side effect similarity

SEs refer to unintended and often undesirable physiological effects that arise from the use of medication. For instance, taking antipyretic analgesics like aspirin may lead to SEs such as heartburn, hives, respiratory difficulties, and impaired kidney function (Blanca-Lopez *et al.* 2017, Weltermann *et al.* 2021). We assumed that if two drugs cause the same SEs, they are likely to have similar functions. Given this assumption, drug similarity was calculated using the Jaccard coefficient in the same way, by evaluating the ratio of common SEs among those associated with each drug.

### 3.4 Weighted-hierarchical similarity between ATC codes

The proposed WHS is measured by applying distinct weights to each hierarchical level of the ATC codes, as shown in Equation (1). Here, k denotes the target level, and $a_{i}$ and $a_{j}$ are the ATC codes for which the similarity is calculated. Equations (2) and (3) are combined in Equation (1).


(1) $${\text{WHS}}_{k}(a_{i},a_{j})=\frac{2W_{k}^{N_{k}(a_{i},a_{j})}+k^{2}}{2W_{k}^{k}+k^{2}},$$

(2) $$N_{k}(a_{i},a_{j})=|S_{k}^{i}\cap S_{k}^{j}|,$$

(3) $$W_{k}^{n}=\{\begin{array}{cc} 0 & \text{if}\;n=0\\ \sum_{i=0}^{n-1}{\left(k-i\right)}^{2} & \text{otherwise} \end{array}$$

In Equation (2), $S_{k}^{i}$ represents the set of codes from the first level to the kth level for $a_{i}$. For example, the fourth-level ATC codes “A03AA” and “A03BB” correspond to the sets $S_{4}^{i}=\{A,A03,A03A,A03AA\}$ and $S_{4}^{j}=\{A,A03,A03B,A03BB\}$, respectively. As a result, the size of their intersection, $N_{4}(a_{i},a_{j})$, is 2.

In Equation (3), weights are assigned to each level of the code, with higher levels receiving higher weights because they represent more specific drug categories. However, the weight increment is adjusted to be less pronounced at higher levels compared to lower levels. Specifically, ATC codes are hierarchically categorized: At the first level, they represent anatomical groups, while the second level codes indicate therapeutic subgroups. The third and fourth levels classify drugs according to their pharmacological and chemical properties. Given this hierarchical structure, it is assumed that the classification differences are greater at lower levels. Therefore, the weight increments are designed to be larger for lower levels and smaller for higher levels.

### 3.5 Masked drug–ATC code associations

Our approach uses known drug–ATC code associations to extract valuable information, not only from drugs associated with the target ATC code but also from other ATC codes linked to the target drug. This information is crucial for predicting ATC codes for drugs, providing a richer context for the model to learn meaningful patterns. However, directly applying these associations without modification is equivalent to relying on the ground truth labels of drug–ATC code pairs, which could lead to overfitting during model training. To mitigate this risk, we employ a masking strategy during the learning process to prevent data leakage. Specifically, we first mask the features of the test pairs in the current fold and then mask the features associated with the target pairs being predicted. This approach ensures that the model is trained without direct exposure to the labels of the test and target pairs, promoting robust and unbiased predictions.

### 3.6 Feature aggregation

The prediction of ATC codes assumes that drugs with similar functions are more likely to have similar codes (Dunkel *et al.* 2008). Considering this assumption, the feature aggregation process combines each type of drug similarity, ATC code similarity, and drug–ATC code association matrices to predict whether the target drug will have a specific code, as illustrated in Fig. 1b.

Each aggregated feature involves two rows: one represents information about the target drug and the other about the target ATC code. The first row contains drug–ATC associations and drug–drug similarities of the target drug, while the second row includes ATC–ATC similarities and ATC–drug associations of the target ATC code. As a result, when the number of drugs is denoted as n and the number of ATC codes at level-k is denoted as $m_{k}$, the final feature has a size of 2×(n+$m_{k}$).

### 3.7 Feature extraction

A convolution layer was introduced to address the loss of spatial and local information that occurs when converting multi-dimensional data to a 1D format for deep neural network training. Using kernels, this layer learns local patterns from small regions of input images or sequence data. As these kernels move across the entire image or sequence data with a defined stride, they effectively extract patterns while preserving the spatial or sequential structure of the data. In our model, a 1D convolution layer is applied to not only capture meaningful local patterns from the aggregated features but also transform them into a format suitable for training a transformer encoder.

Transformer is a model designed to leverage the self-attention mechanism, overcoming the constraints of the recurrent neural network (RNN) in parallel processing and achieving high performance in natural language processing tasks. By allowing each component of the incoming data to independently calculate its relationship with other components, self-attention significantly increases processing speed and eliminates reliance on sequential processing. As a result, transformer can learn more efficiently than RNN.

In this study, as described in Fig. 1c, we adopted the encoder component of the transformer, called the TR block. The encoder primarily consists of multi-head self-attention and a feed-forward neural network. Multi-head self-attention employs multiple attention heads, independently generating queries (Q), keys (K), and values (V). These are processed in parallel to compute attention. This process can be expressed as follows:


(4) $$\text{Attention}(Q,K,V)=\text{softmax}\left(\frac{QK^{T}}{\sqrt{d_{k}}}\right)V,$$

where $d_{k}$ represents a scaling factor introduced to enhance the stability of the training process. Through this mechanism, each attention head learns to capture the relationships between elements, and the combined outputs from multiple attention heads are aggregated to compute the final attention. The resulting attention from the combination of all heads is expressed as follows:


(5) $$Z=[{\text{Attention}}_{1};\ldots ;{\text{Attention}}_{h}]$$

Let h denote the number of parallel attention heads. The attention information obtained through this process effectively captures the contextual elements of the entire dataset. Subsequently, the feed-forward neural network comprises two linear transformations interleaved with a nonlinear activation function. The activation function introduces nonlinearity, enabling the model to learn complex patterns in the input data more effectively. This architecture can contribute to the accurate prediction of associations between drugs and ATC codes.

### 3.8 Prediction

As shown in Fig. 1d, three final embeddings derived from the three drug similarity matrices are concatenated and then compressed into a single representation through a fully connected layer. Subsequently, this representation is passed through a sigmoid layer to estimate the association probability of the target drug–ATC code pair.

## 4 Experiments and results

### 4.1 Datasets

We first retrieved CSs, DDIs, and ATC codes for the drugs from DrugBank (Knox *et al.* 2024). In addition, drug SE data were obtained from the SIDER database (Kuhn *et al.* 2016). From these datasets, we acquired CSs for 2841 drugs, 238 types of DDIs, and 5880 SE records for use in our experiments. The associations between drugs and ATC codes were established based on the hierarchical structure of the ATC codes. Table 1 provides a summary of the data statistics for each level. The experiments were conducted using levels 1 through 4, as level 5 consists of unique designations for each code, which were excluded from analysis. Negative sampling was also applied to guarantee balanced training, resulting in a final dataset with an equal distribution of positive and negative pairs.

**Table 1.** The total number of positive drug–ATC code pairs and the number of distinct ATC codes at each hierarchical level.

Level	Positive pairs	Categories of ATC code
1	3348	14
2	3509	86
3	3772	230
4	4013	698

### 4.2 Performance evaluation metrics

Our binary classification model predicts whether the target drug–ATC code pair is associated. In this study, we employ two evaluation metrics, the area under the receiver operating characteristic curve (AUROC) and the area under the precision-recall curve (AUPRC), to quantitatively assess and compare the predictive performance of our model against benchmark models. AUROC measures the area under the ROC curve which plots the true positive rate against the false positive rate. AUPRC, on the other hand, measures the area under the precision-recall curve which plots precision against recall at various threshold settings.

We evaluated the model’s capacity for generalization using a 10-fold cross-validation. The dataset consists of ten folds, nine of which are used for training and the other for validation. This cross-validation process is conducted on each of the ten folds to verify whether the model overfits to a particular subset of the data and to ensure consistent performance across various splits of the dataset. By computing the mean and standard deviation of prediction performance over 10 folds, the performance table that follows is created, which consequently allows us to evaluate whether the model produces stable and reliable predictions across a range of datasets.

### 4.3 Performance comparison

In this study, we evaluated the performance of our proposed model by comparing it against state-of-the-art models for predicting drug–ATC code associations through binary classification. To ensure a comprehensive evaluation, the results were compared from the first to the fourth levels of ATC codes, as described in Tables 2 and 3. The models considered for comparison include traditional machine learning algorithms such as k-nearest neighbors (KNN) and random forest (RF), as well as high-performance models from recent studies, namely NetPredATC, SPACE, and RNPredATC. KNN was configured with $n_{\text{neighbors}}=5$, and RF was set with $n_{\text{estimators}}=100$. NetPredATC employs an SVM and combines drug and ATC code similarities using a kernel technique to predict the relationships between drugs and ATC codes. CS similarity was applied to compute drug similarities, following the methodology outlined in the original research. SPACE uses a variety of features, including CS similarity, target protein similarity, SE similarity, gene expression similarity, and compound association similarity. These features are selected using the minimum redundancy maximum relevance method (Ding and Peng 2005), and predictions are performed using logistic regression. In this experiment, the SPACE model was trained using similarities derived from CS and SE information. Finally, RNPredATC constructs feature matrices that include drug similarity, ATC code similarity, and drug–ATC code association, and applies deep residual learning for association prediction. Considering the model structure and hyperparameter values outlined in the original work, RNPredATC was trained using CS similarity.

**Table 2.** AUROC results of the proposed model, FACT, and comparative methods in ATC code prediction.

	Level 1	Level 2	Level 3	Level 4
**RF**	0.7594 ± 0.0189	0.7357 ± 0.0248	0.7286 ± 0.0207	0.6624 ± 0.0392
**KNN**	0.7492 ± 0.0244	0.7726 ± 0.0194	0.7855 ± 0.0127	0.7495 ± 0.0176
**NetPredATC**	0.7451 ± 0.0676	0.7594 ± 0.0146	0.7686 ± 0.0182	0.7532 ± 0.0197
**SPACE**	0.9236 ± 0.0211	0.9221 ± 0.0212	0.8879 ± 0.0176	0.8300 ± 0.0202
**RNPredATC**	0.8126 ± 0.0980	0.9631 ± 0.0300	0.8417 ± 0.0757	0.8011 ± 0.0690
**FACT**	**0.9803** ± **0.0065**	**0.9825** ± **0.0043**	**0.9829** ± **0.0051**	**0.9805** ± **0.0064**

The best results are written in bold and the second-best results are underlined.

**Table 3.** AUPRC results of the proposed model, FACT, and comparative methods in ATC code prediction.

	Level 1	Level 2	Level 3	Level 4
**RF**	0.7659 ± 0.0201	0.7630 ± 0.0250	0.7355 ± 0.0223	0.6801 ± 0.0420
**KNN**	0.7561 ± 0.0245	0.7760 ± 0.0207	0.7945 ± 0.0146	0.7669 ± 0.0164
**NetPredATC**	0.7093 ± 0.0709	0.6803 ± 0.0161	0.6791 ± 0.0127	0.6625 ± 0.0183
**SPACE**	0.8740 ± 0.0211	0.8906 ± 0.0284	0.8462 ± 0.0249	0.7869 ± 0.0273
**RNPredATC**	0.8030 ± 0.0904	0.9502 ± 0.0387	0.8309 ± 0.0704	0.7928 ± 0.0536
**FACT**	**0.9773** ± **0.0081**	**0.9818** ± **0.0043**	**0.9812** ± **0.0065**	**0.9770** ± **0.0072**

The best results are written in bold and the second-best results are underlined.

Tables 2 and 3 present AUROC and AUPRC results, respectively, as the model’s predictive performance across different ATC levels. The tables present the mean and standard deviation of AUROC and AUPRC across all folds. Both AUROC and AUPRC results indicate that traditional machine learning models, such as KNN and RF, as well as the NetPredATC model, exhibit significantly lower performance compared to the recently proposed SPACE and RNPredATC models across all levels. Although the predictive performance of the SPACE and RNPredATC models ranged from 0.8011 to 0.9631 in terms of AUROC and 0.7869 to 0.9503 in terms of AUPRC, performance at level 4 was significantly lower than that at levels 1, 2, and 3. In contrast, our proposed model, FACT, consistently outperformed all comparative models at every ATC level, maintaining robust and high predictive performance. Specifically, FACT showed high accuracy and low variance across levels with AUROC values ranging from 0.9803 to 0.9829 and AUPRC values ranging from 0.9770 to 0.9818. This indicates that FACT effectively analyzes drug–ATC associations through feature extraction process. Additionally, even at level 4, the model exhibits consistent performance, highlighting its potential as the most reliable method for predicting drug ATCs and overcoming the drawbacks of earlier models.

### 4.4 Impact of feature combinations on prediction performance

**Figure 2.**
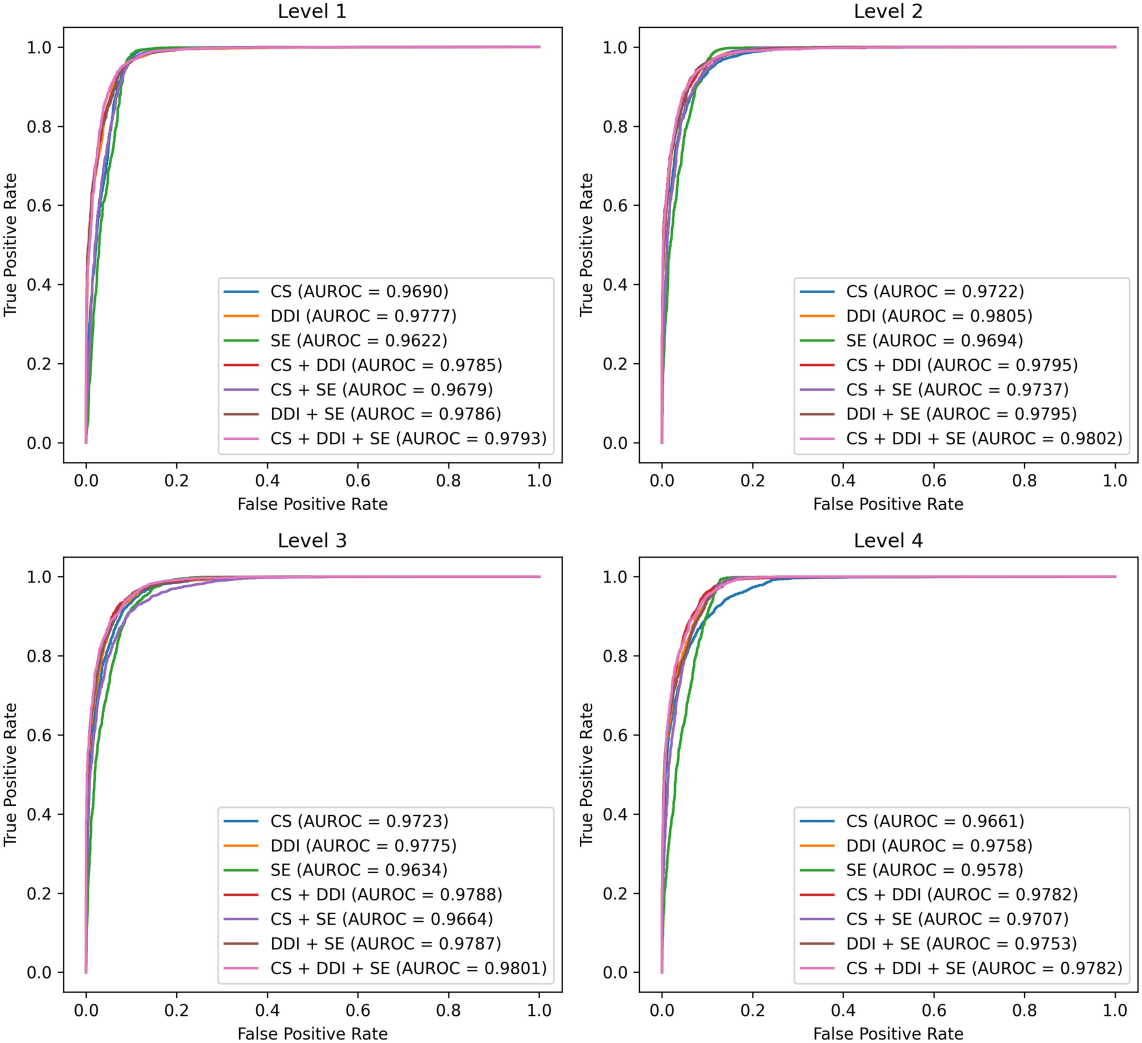
ROC curves and AUROC results of FACT across all drug feature combinations in ATC code prediction.

**Figure 3.**
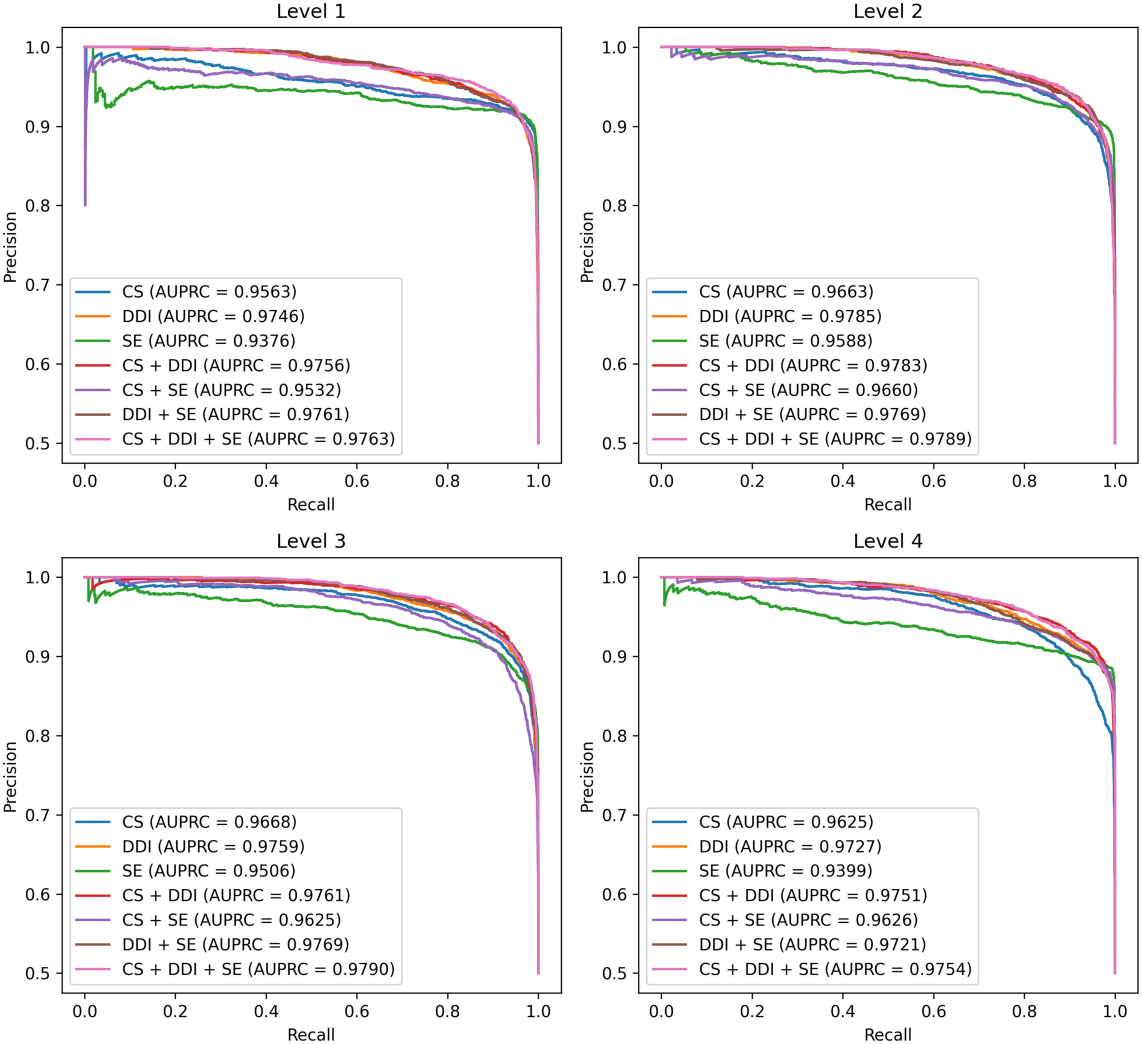
Precision-recall curves and AUPRC results of FACT across all drug feature combinations in ATC code prediction.

We conducted additional experiments to assess how different combinations of drug features impact the prediction performance for ATC classification. Figures 2 and 3 present the ROC and PR curves for each set of feature matrices, generated from various levels of CS similarity, DDI similarity, and drug SE similarity. According to the results derived from a single feature, DDI achieved the highest AUROC and AUPRC, followed by CS and SE, across all levels. Notably, at level 4, the AUROC and AUPRC values for DDI were 0.9758 and 0.9727, respectively, surpassing the AUROC and AUPRC values of 0.9661 and 0.9625 for CS and 0.9578 and 0.9399 for SE. This indicates that DDI provides more significant information for predicting ATC codes than the other two features. This trend was also observed in models combining two features. Experimental results revealed that models using a greater number of features generally showed improved prediction performance, with those incorporating DDI outperforming models that did not, such as the SE + CS model. This result further strengthens our view that DDI provides the most crucial information for prediction. Finally, the model using all three proposed features demonstrated performance improvements at every level, obtaining the AUROC of up to 0.9802 and the AUPRC of up to 0.9790, confirming an impressive increase in prediction accuracy. FACT gained a deeper understanding of the drugs by incorporating a variety of drug similarity data, which eventually led to superior performance in the ATC code prediction task.

### 4.5 Evaluation of ATC code similarity

In this section, we conducted comparative analyzes between our proposed similarity formula and two existing methods. NetPredATC calculates ATC code similarity following the procedure described in Equation 6. This approach calculates the weight of an ATC code $a_{i}$, represented as $w(a_{i})$, as the inverse of the proportion of positive samples that $a_{i}$ occupies a given level. Additionally, $d(a_{i},a_{j})$ represents the distance between two ATC codes, $a_{i}$ and $a_{j}$, within the hierarchical structure, capturing their dissimilarity. In the RNPredATC model, the similarity is computed by the intersection of the subsets, which measures how many hierarchical levels the two ATC codes have in common, as shown in Equation 7. They are given equal weights at all levels. For level 1, where only one code is predicted, the result is the same as in our model. However, the RNPredATC model applies the same weights to all levels, whereas our model, WHS, assigns weights based on level-specific attributes for levels 2, 3, and 4.


(6) $${\text{NP}}_{\text{Sim}}(a_{i},a_{j})=w(a_{i})w(a_{j})\;\text{exp}\;\left(-0.25\cdot d(a_{i},a_{j})\right)$$

(7) $${\text{RNP}}_{\text{Sim}}^{k}(i,j)=\frac{2\cdot |S_{k}^{i}\cap S_{k}^{j}|+1}{2k+1}$$

As shown in Tables 4 and 5, the experimental results revealed that using ${\text{NP}}_{\text{Sim}}$ resulted in relatively lower performance compared to the other two similarity formulas. Specifically, WHS achieved improvements over ${\text{NP}}_{\text{Sim}}$ by 1.81%–5.27% in AUROC and 1.28%–4.64% in AUPRC at all levels. Although the outcomes of ${\text{RNP}}_{\text{Sim}}$ and WHS were identical at level 1 due to the lack of weights in WHS, ${\text{RNP}}_{\text{Sim}}$ showed marginally higher AUPRC at level 3 over WHS, with values of 0.9808 and 0.9805, respectively. In other cases, WHS demonstrated better performance than ${\text{RNP}}_{\text{Sim}}$. These results affirm the robustness of our method in effectively capturing hierarchical relationships in ATC code prediction.

**Table 4.** AUROC results of FACT with different ATC code similarity formulations.

	${\text{NP}}_{\text{Sim}}$	${\text{RNP}}_{\text{Sim}}$	WHS
Level 1	0.9276 ± 0.0122	**0.9803** ± **0.0065**	**0.9803** ± **0.0065**
Level 2	0.9406 ± 0.0136	0.9819 ± 0.0053	**0.9825** ± **0.0043**
Level 3	0.9386 ± 0.0174	0.9818 ± 0.0052	**0.9820** ± **0.0054**
Level 4	0.9624 ± 0.0175	0.9785 ± 0.0077	**0.9805** ± **0.0064**

The best results are written in bold and the second-best results are underlined.

**Table 5.** AURRC results of FACT with different ATC code similarity formulations.

	${\text{NP}}_{\text{Sim}}$	${\text{RNP}}_{\text{Sim}}$	WHS
Level 1	0.9309 ± 0.0126	**0.9773** ± **0.0081**	**0.9773** ± **0.0081**
Level 2	0.9453 ± 0.0104	0.9806 ± 0.0057	**0.9818** ± **0.0043**
Level 3	0.9449 ± 0.0164	**0.9808** ± **0.0062**	0.9805 ± 0.0064
Level 4	0.9642 ± 0.0160	0.9748 ± 0.0102	**0.9770** ± **0.0072**

The best results are written in bold and the second-best results are underlined.

### 4.6 Hyperparameter optimization

**Figure 4.**
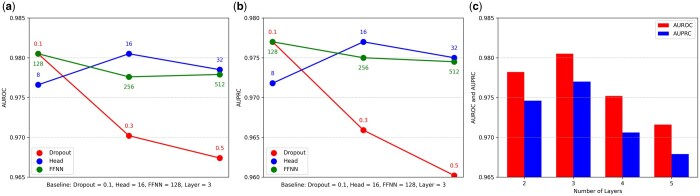
Exploration of hyperparameter settings for optimal model performance. (a) and (b) The results of modifying dropout rate, the number of attention heads, and the hidden dimension of the feed-forward neural network, evaluated using AUROC and AUPRC, respectively. (c) The impact of modifying the number of TR blocks.

To optimize the model, we conducted a series of experiments by tuning various hyperparameters: the dropout rate, the number of attention heads, the hidden dimension of the feed-forward neural network in the TR block, and the number of TR blocks. As shown in Fig. 4, each plot represents the performance comparison when one hyperparameter is varied from the baseline, which yields optimal performance. For the dropout rate, optimal AUROC and AUPRC values of 0.9805 and 0.9770, respectively, were achieved at a rate of 0.1. As the dropout rate increased, both performance metrics showed a decline, with AUROC and AUPRC dropping to 0.9702 and 0.9659 at a rate of 0.3 and further to 0.9674 and 0.9602 at 0.5. The number of attention heads yielded the best performance at 16, with AUROC and AUPRC values of 0.9785 and 0.9750 at 32, and 0.9766 and 0.9718 at 8. Similarly, for the feed-forward neural network’s hidden dimensions, optimal performance was observed at 128, with AUROC and AUPRC of 0.9776 and 0.9750 at 256, and 0.9779 and 0.9745 at 512. Lastly, FACT showed the best performance using 3 TR blocks. With 2 layers, performance dropped to 0.9782 and 0.9746; with 4 layers, it decreased further to 0.9752 and 0.9706; and with 5 layers, the performance was 0.9716 and 0.9679. These results suggest a slight decrease in performance when the number of layers exceeds 3. Additional hyperparameter configurations, as well as details regarding training time and hardware requirements, are provided in Supplementary Materials S1 and S2, respectively.

### 4.7 Robustness test

To assess the robustness of FACT, we conducted a series of perturbation experiments by systematically modifying the training data. Specifically, the experiments consisted of two types: false negative injection and false positive injection. The first involved deliberately injecting known drug–ATC pairs into the negative training set, while the second added unverified pairs into the positive training set. These experimental approaches allowed us to evaluate both the robustness of FACT and the reliability of its negative sampling strategy.

As shown in Table 6, we tested FACT under dataset perturbations of ±10% and ±20%. As a result, FACT demonstrated consistently high performance across all perturbation scenarios, with only marginal fluctuations—ranging from 0.05% to 1.26% in terms of AUROC and from 0.26% to 1.64% in terms of AUPRC. Overall, these results confirm that FACT maintains stable predictive performance under realistic data imperfections, highlighting its robustness.

**Table 6.** Robustness test results of FACT. –20% and −10% represent scenarios where known positives were intentionally mislabeled as negatives, while +10% and +20% indicate adding potentially noisy positives into the positive set.

Metric	Level	−20%	−10%	Original	+10%	+20%
AUROC	1	0.9679 ± 0.0108	0.9751 ± 0.0062	0.9803 ± 0.0065	0.9761 ± 0.0078	0.9774 ± 0.0056
	2	0.9742 ± 0.0099	0.9774 ± 0.0071	0.9825 ± 0.0043	0.9784 ± 0.0087	0.9792 ± 0.0074
	3	0.9779 ± 0.0071	0.9817 ± 0.0057	0.9829 ± 0.0051	0.9814 ± 0.0063	0.9824 ± 0.0054
	4	0.9720 ± 0.0103	0.9758 ± 0.0094	0.9805 ± 0.0064	0.9758 ± 0.0074	0.9749 ± 0.0090
AUPRC	1	0.9612 ± 0.0129	0.9685 ± 0.0087	0.9773 ± 0.0081	0.9728 ± 0.0093	0.9730 ± 0.0084
	2	0.9706 ± 0.0099	0.9738 ± 0.0087	0.9818 ± 0.0043	0.9763 ± 0.0096	0.9774 ± 0.0082
	3	0.9754 ± 0.0087	0.9785 ± 0.0089	0.9812 ± 0.0065	0.9784 ± 0.0080	0.9786 ± 0.0085
	4	0.9661 ± 0.0124	0.9713 ± 0.0113	0.9770 ± 0.0072	0.9723 ± 0.0085	0.9712 ± 0.0101

### 4.8 Complementary studies

To further demonstrate the practical applicability and interpretability of FACT, we conducted a series of additional experiments. First, we performed a case study on drug–ATC code prediction to evaluate the real-world relevance of the predictions. The case study results are provided in Supplementary Material S3. Second, we analyzed the model’s interpretability to better understand the contribution of each input feature (CS, DDI, and SE). The detailed interpretability analysis is provided in Supplementary Material S4.

## 5 Conclusion and discussion

Predicting ATC codes for drugs can be a valuable task in drug repositioning, potentially leading to improved outcomes. In this study, we propose FACT, a unique framework that utilizes three types of intrinsic attributes of drugs: CS, DDIs and SEs. By calculating drug similarities from these sources, FACT constructs three distinct feature matrices, aggregating the WHS of ATC codes and masked drug–ATC associations. These matrices are then processed using a CT encoder that incorporates convolution and TR blocks, enabling FACT to capture intricate correlations between drugs and ATC codes.

Experimental results demonstrate that FACT outperforms existing methods across all ATC hierarchies, highlighting its ability to predict relationships between drugs and ATC codes. Furthermore, additional experiments reveal that DDI data plays an important role in the prediction of ATC codes, and incorporating additional drug-related data further improves performance. Notably, FACT achieves high predictive performance even when using individual features alone, demonstrating the contribution of the usage of the masking approach and WHS in FACT architecture. The WHS of ATC codes also contributes to a slight improvement, surpassing traditional similarity measurement approaches. Hyperparameter optimization reveals that FACT achieves optimal performance with a dropout rate of 0.1, 16 attention heads, a hidden dimension of 128 in the feed-forward network, and 3 TR blocks, resulting in AUROC and AUPRC scores of 0.9805 and 0.9770, respectively.

Despite its high predictive performance, our model has several limitations. First, the negative sampling technique used to generate training examples can inadvertently include positive pairs that remain unidentified, as with other models. Second, the model’s ability to predict ATC codes for new drugs without prior associations is constrained. Therefore, regular updates incorporating new drugs and data are essential to maintain its reliability. Third, this study lacks validation on an independent external dataset due to limited data availability. Future studies should aim to validate the model using external databases to further demonstrate generalizability. Lastly, this study used fingerprints to compute chemical structural similarity, which may overlook critical 3D structural details of drugs. To overcome this limitation, future research should explore ways to integrate three-dimensional coordinates and graph structures.

## Supplementary Material

btaf184_Supplementary_Data

## Data Availability

The code and data are available at https://github.com/knhc1234/FACT.
