# Supplementary material for: FACT: Feature Aggregation and Convolution with Transformers for predicting drug classification code
Source: Bioinformatics. 2025 Jul 15;41(Suppl 1):i77–85. doi: 10.1093/bioinformatics/btaf184 (PMC12261408; doi:10.1093/bioinformatics/btaf184)
Supplement: btaf184_Supplementary_Data [file btaf184_supplementary_data.pdf]

**Supplementary Information for:**

# FACT: Feature Aggregation and Convolution with Transformers for predicting drug classification code

Gwang-Hyeon Yun[1], Jong-Hoon Park[1] and Young-Rae Cho[1,2,*]

[1]Division of Software, Yonsei University - Mirae Campus, 1 Yeonsedae-gil, Wonju-si, Gangwon-do 26493, Republic of Korea

[2]Division of Digital Healthcare, Yonsei University - Mirae Campus, 1 Yeonsedae-gil, Wonju-si, Gangwon-do 26493, Republic of Korea

*Corresponding author: youngcho@yonsei.ac.kr

## Supplementary 1. Parameter Setting.

Table S1. Hyperparameters for the convolution layer and transformer encoder layer.

| Convolution Layer | | Transformer Encoder Layer | |
|---|---|---|---|
| kernel size | 3 | input dimension | 128 |
| stride | 2 | dropout rate | 0.1 |
| padding | 1 | number of multi-heads | 16 |
| output dimension | 128 | feed-forward dimension | 128 |

The parameter settings used to ensure the repeatability of the experiments are described above. The Adam optimizer and the binary cross-entropy loss were employed in the training procedure. The batch size was set to 128, and the learning rate was assigned as 0.0005. Each fold had a total of 50 epochs to set up, and early stopping was applied to prevent overfitting if performance did not improve after 10 consecutive epochs.

Table S1 presents the hyperparameter settings for the model. The convolution layer was configured with a kernel size of 3 to capture local patterns in the input data effectively. The stride was 2 to reduce the spatial dimensions, while padding was configured at 1 to minimize information loss at the boundaries. The output dimension was 128 to enhance the representational capacity of the extracted features.

In order to balance representational capacity and computational efficiency across layers, the hyperparameters of the transformer encoder layer were set as follows: the model dimension was assigned as 128. To enhance generalization and avoid overfitting, the dropout rate was set at 0.1. The number of attention heads was configured at 16, enabling the model to effectively learn relationships across various subspaces of the input data. Furthermore, to optimize each layer's capacity for data processing, the feed-forward network dimension was set to 128.

## Supplementary 2. Computational Efficiency and Scalability

Table S2. Model complexity and training time of FACT.

| level | batch size | # Parameters | GPU memory Usage | Time (1 epoch) | Training Time (50epoch x 10Fold) |
|---|---|---|---|---|---|
| 1 | 128 | 1,198,081 | 11.644GB | 70 sec | 35,000 sec ≈ 9.7 hours |
| 2 | 128 | 1,198,081 | 11.940GB | 74 sec | 37,000 sec ≈ 10.3 hours |
| 3 | 128 | 1,198,081 | 12.402GB | 87 sec | 43,500 sec ≈ 12.1 hours |
| 4 | 128 | 1,198,081 | 14.304GB | 124 sec | 62,000 sec ≈ 17.2 hours |

Table S2 represents the model's complexity in terms of the number of parameters, GPU memory usage, and training time. The FACT model consists of 1,198,081 parameters, regardless of the ATC level. The model structure remains consistent across all experiments, ensuring stable computational demands. Moreover, the current version of FACT has been validated on datasets consisting of 2,841 drugs and up to 698 ATC categories. While scaling FACT to significantly larger datasets presents a potential challenge, its efficient design — characterized by a moderate number of parameters and manageable GPU memory requirements — makes it well-suited for larger datasets when using appropriate hardware (e.g., multi-GPU systems). Future work will explore optimizing FACT for distributed computing environments to efficiently handle drug databases comprising millions of compounds.

## Supplementary 3. Analysis of Prediction Results

Table S3. Top 10 drug-ATC pairs without known associations, but predicted as related by FACT.

| DrugBank ID | Drug Name | Original ATC Code | Predicted ATC Code |
|---|---|---|---|
| DB11699 | Tropisetron | A04AA | D05AC |
| DB00248 | Cabergoline | N04BC, G02CB | C03AX |
| DB00213 | Pantoprazole | A02BC, A02BD | B03AD |
| DB01606 | Tazobactam | J01CG | B03BA |
| **DB14533** | **Zinc chloride** | **B05XA, C05AX** | **R05CB** |
| DB00463 | Metharbital | N03AA | D08AG |
| DB00829 | Diazepam | N05BA | H03AA |
| DB00441 | Gemcitabine | L01BC | D06AX |
| **DB11748** | **Benfotiamine** | **A11DA** | **B03BA** |
| DB13762 | Dexrabeprazole | A02BC | A01AC |

To evaluate whether our model can be effectively utilized for drug repurposing, we analyzed the top 10 drug-ATC code predictions with the highest prediction scores when previously unannotated drug-ATC pairs were input into the trained model (Table S3). Among these, we selected two representative cases—Zinc chloride and Mucolytic Agents (R05CB), and Benfotiamine and vitamin B12 analogs (B03BA)—for further analysis.

Case 1: Zinc Chloride and Mucolytic Agents(R05CB)

Zinc chloride ($ZnCl_2$) is currently classified under C05AX (other varicose vein treatment preparations) and B05XA (electrolyte solutions). However, FACT predicts that $ZnCl_2$ may functionally resemble R05CB (mucolytics), suggesting a potential role in respiratory therapy beyond its current indications. Specifically, Zn2+ inhibits the NF-kB pathway, reducing inflammatory mucus secretion and regulating airway inflammation [1]. $ZnCl_2$ also enhances antioxidant defense mechanisms in respiratory epithelial cells by promoting the expression of SOD1/GPX1 genes, thereby reducing oxidative damage [2]. Clinical studies have shown that zinc chloride-containing mouthwashes are effective in preventing mucositis in chemotherapy patients, supporting its role in mucosal tissue regeneration [3]. Unlike typical mucolytics, which directly break down mucus, zinc chloride may exert complementary effects through its anti-inflammatory and protective properties. Based on this complementary mechanism, $ZnCl_2$ may serve a similar therapeutic role to mucolytics by modulating mucus and offering respiratory protection.

Case 2: Benfotiamine and vitamin B12 analogs(B03BA)

FACT predicted a functional similarity between Benfotiamine (A11DA03) and vitamin B12 analogs (B03BA), suggesting a potential overlap in their neuroprotective properties. Although the B03BA category primarily includes drugs related to hematopoietic and blood-related functions, vitamin B12 also plays a crucial role in maintaining neural function and treating peripheral neuropathies, which may have contributed to the predicted similarity with Benfotiamine. In fact, Benfotiamine is also involved in neuroprotection and energy metabolism regulation, particularly in the treatment of diabetic neuropathy, highlighting a common therapeutic application with vitamin B12 [4], [5], [6]. Despite differences in their mechanisms of action with Benfotiamine enhancing carbohydrate metabolism and neuronal energy supply, while vitamin B12 is essential for methylation reactions and myelin synthesis, both compounds share neuroprotective functions. This suggests that FACT's prediction may be useful for identifying potential therapeutic similarities between ATC-coded drugs, highlighting its applicability in drug repurposing research and its potential to uncover previously unrecognized functional relationships among existing pharmaceuticals.

References

[1] Luan, R., Ding, D., Xue, Q., Li, H., Wang, Y., and Yang, J. (2023). Protective role of zinc in the pathogenesis of respiratory diseases. *European Journal of Clinical Nutrition*, 77(4):427–435.

[2] Salesa, B., Serra, R. S. I., and Ángel Serrano-Aroca (2021). Zinc chloride: Time-dependent cytotoxicity, proliferation and promotion of glycoprotein synthesis and antioxidant gene expression in human keratinocytes. *Biology (Basel)*, 10(11):1072.

[3] Oshvandi, K., Vafaei, S. Y., Kamallan, S. R., Khazaei, S., Ranjbar, H., and Mohammadi, F. (2021). Effectiveness of zinc chloride mouthwashes on oral mucositis and weight of patients with cancer undergoing chemotherapy. *BMC Oral Health*, 21(1):364.

[4] Baltrusch, S. (2021). The role of neurotropic b vitamins in nerve regeneration. *BioMed Research International*, 2021:9968228.

[5] Tapias, V., Jainuddin, S., Ahuja, M., Stack, C., Elipenahli, C., Vignisse, J., Gerges, M., Starkova, N., Xu, H., Starkov, A. A., Bettendorff, L., Hushpulian, D. M., Smirnova, N. A., Gazaryan, I. G., Kaidery, N. A., Wakade, S., Calingasan, N. Y., Thomas, B., Gibson, G. E., Dumont, M., and Beal, M. F. (2018). Benfotiamine treatment activates the Nrf2/ARE pathway and is neuroprotective in a transgenic mouse model of tauopathy. *Human Molecular Genetics*, 27(16):2874–2892.

[6] Calderón-Ospina, C. A. and Nava-Mesa, M. O. (2020). B vitamins in the nervous system: Current knowledge of the biochemical modes of action and synergies of thiamine, pyridoxine, and cobalamin. *CNS Neuroscience Therapeutics*, 26(1):5–13.

## Supplementary 4. SHAP-based Feature Importance and Model Interpretability Analysis

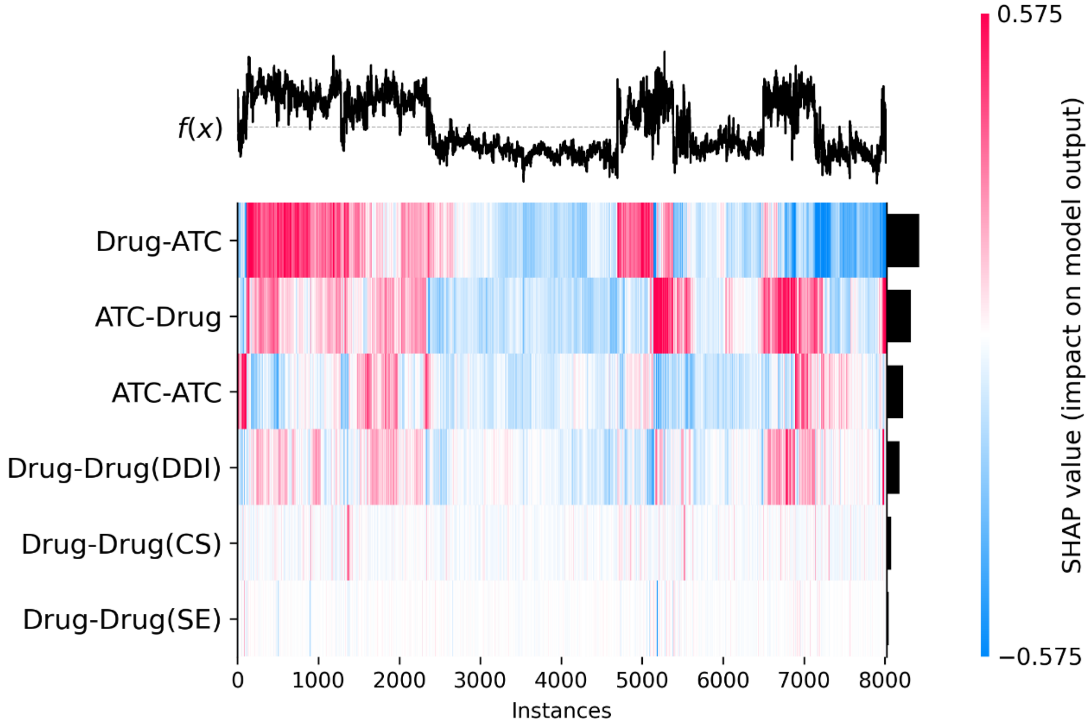


Figure S1. SHAP-based heatmap of feature importance. Drug-ATC indicates the associations between the target drug and all ATC codes, while ATC-Drug represents the associations between the target ATC code and all drugs. ATC-ATC denotes the similarity between ATC codes, and Drug-Drug rows correspond to drug-drug similarity calculated by each method: CS (chemical structure), DDI (drug-drug interaction) and SE (side effect).

To enhance the interpretability of the FACT model's predictions, we conducted a feature importance analysis using SHAP (SHapley Additive exPlanations). This approach quantifies the relative contribution of each feature to the model's output f(x), providing insights into how the FACT model integrates various data sources to make predictions.

As illustrated in Figure S1, the features representing the relationships between drugs and ATC codes (Drug-ATC and ATC-Drug) exhibited the highest SHAP values. This indicates that the FACT model effectively learns and utilizes cross-domain associations between heterogeneous information types, specifically drug properties and ATC classifications, to improve its predictive performance in drug-ATC code association tasks.

Following ATC-ATC similarities, the DDI-based drug similarities also demonstrated substantial contributions to the model's predictions. This finding aligns with the feature ablation study results presented in the main text, further confirming that while DDI information is an important factor, ATC similarity plays a more dominant role in the FACT model's decision-making process.

Conversely, drug-drug similarities derived from CS and SE exhibited relatively lower SHAP values, suggesting that these types of information played a more supplementary role in the prediction process. These results imply that while the FACT model is designed to incorporate a diverse range of feature types, it primarily leverages functional and clinical associations to drive its predictive capabilities.
